# Bridging healthcare gaps through specialized mobile healthcare services to improve healthcare access and outcomes in rural Hungary

**DOI:** 10.1038/s41598-025-97447-9

**Published:** 2025-04-12

**Authors:** Mark Virag, Rita Kovacs, Gergely Marovics, Luca Toth, Barbara Sandor, Peter Voros, Veronika Gyori–Dani, Ferenc Nagy, Daniel Eorsi, Janos Sandor, Istvan Kiss, Ferenc Vincze, Anita Palinkas, Abel Perjes, Szilard Rendeki, Peter Maroti

**Affiliations:** 1 Medical School, Department of Public Health Medicine, University of Pecs, Szigeti str. 12, HU–7624 Pecs, Hungary; 2 Hungarian Charity Service of the Order of Malta, Fortuna str. 10, Budapest, H–1014 Hungary; 3 Medical School, Clinical Centre, Department of Neurosurgery, University of Pecs, Ret str. 2, Pecs, HU–7624 Hungary; 4 Medical School, Clinical Centre, 1st Department of Medicine, Division of Preventive Cardiology and Rehabilitation, University of Pecs, Pecs, HU–7624 Hungary; 5 Faculty of Medicine, Department of Public Health and Epidemiology, University of Debrecen, Kassai str. 26, Debrecen, HU–4028 Hungary; 6 Medical School, Medical Skills Education and Innovation Centre, Universitys of Pecs, Szigeti str. 12, Pecs, HU–7624 Hungary; 7 Medical School, 3D Printing and Visualization Centre, University of Pecs, Boszorkany str. 2, Pecs, HU–7624 Hungary; 8 Doctoral School of Mental Health Sciences, Semmelweis University, 25 Üllői Str, Budapest, HU-1091 Hungary

**Keywords:** Telemedicine, Rural healthcare access, Mobile health clinics, Telemedicine ecosystem, Underserved populations, Point-of-care technology, Health policy, Health services, Public health, Information technology

## Abstract

Telemedicine offers promising solutions to healthcare challenges in underserved rural areas. This study evaluates a public insurance-based telemedicine system implemented in Hungary, where 12 Mobile Healthcare Service Centers (MHSCs) provided general and specialist care to over 21,000 residents across five rural districts. During the six-month study period, 1,889 individuals received medical care through 4,118 healthcare events. The intervention led to the identification of 105 new hypertension cases and 26 new diabetes cases. Patient feedback was overwhelmingly positive, with 96% willing to recommend the service, and nearly all doctors expressing a willingness to continue participation. A 10% reduction in relative referral rates was observed, suggesting that the system helped alleviate pressure on traditional healthcare providers. Additionally, 2,026 screenings, 1,572 chronic care visits, and 151 laboratory tests were conducted, demonstrating the broad utilization of the service. The estimated monthly operational cost of the telemedicine system was approximately $250,000, which exceeded the national primary healthcare funding of $160,000 per month, highlighting the financial implications of scaling similar programs. The findings indicate that mobile telemedicine services can improve healthcare accessibility in underserved areas and reduce healthcare disparities. Further research is needed to assess the long-term effects of such interventions.

**Supplementary Information:**

The online version contains supplementary material available at 10.1038/s41598-025-97447-9.

## Introduction

Digital healthcare is defined as the use of digital technologies for health purposes, and it is considered as an umbrella term that involves mHealth (mobile health), eHealth (electronic health) and the use of computer science, including areas like big data or artificial intelligence^1,2^. Digital healthcare technologies have undergone significant and remarkable improvements in the past decades, thanks to the continuous development of information technology (IT), medical instrumentation and the wider availability of broadband connection and digital networks on a global scale^1–3^. Telemedicine, as part of digital healthcare, is defined as “*the use of information and communication technologies to improve patient outcomes by increasing access to care and medical information*” according to WHO (World Health Organization), while the definition of Telehealth is considered as the “*the most basic engagement of eHealth*,* involving telecommunications and virtual technology to deliver health care outside of traditional facilities*.” The American Telemedicine Association uses the two terms as synonyms^1^.

As the definitions highlight, telemedicine plays a crucial role in the support of healthcare services outside clinical centers, hospitals or other conventional medical infrastructure. The so called “medical deserts” – such as rural and remote areas – have limited access to qualified healthcare providers and quality healthcare services^4^. The circumstances in which people are born, grow, live, or work strongly determinates the access and quality of medical care. The unique challenges faced by rural populations, such as limited access to healthcare services, higher prevalence of chronic conditions, and poorer health outcomes compared to urban areas underscore the importance of improving telemedicine solutions serving those “medical deserts”^5^.

The use of technology to deliver healthcare services across the spectrum of primary secondary and tertiary care has globally improved access to healthcare services^6,7^.

Mobile health clinics like specially equipped motor vehicles, that travel to various communities to provide healthcare services directly to people who may have limited access to traditional healthcare facilities, showed promising results in nationwide delivery of primary care and screening activities. The analysis of mobile clinics indicated that they can be cost–effective, particularly in preventive care^5–7^. A recent study conducted in the United States – analyzing 1,498 patient encounters – reported, that mobile health clinics (MHC) can replace institutional healthcare, and MHC was found as an effective solution for rural healthcare crisis^5^.

Based on the currently published literature, telemedicine has been particularly beneficial for those who are geographically deprived or cannot easily access healthcare services. Consequently, telemedicine can be regarded as a highly beneficial to address health disparities^8^. Previous studies on the field agree, that digital healthcare technologies involving telemedicine–based care can get control of the abovementioned challenges, reducing healthcare cost, improve patient outcomes, satisfaction, and can potentially overcome geographical barriers as well^9–12^. Subsets of telemedicine include teleradiology, teleneurology, teledermatology, telecardiology, teleophthalmology, telestroke, telerehabilitation^13^, teleoncology, teledentistry, and telepsychiatry applications as well^13–21^.

From the technical aspect, telemedicine–based care can potentially involve teleconsultation using telecommunications and audiovisual systems, telemonitoring with smart or mobile devices and POCT systems. Early studies on telemedicine suggest that, distributed laboratory settings can be cost–effective and adequate alternatives compared to centralized institutes^11^.

In the management of chronic diseases, telemedicine offers numerous solutions in prevention, diagnostics, monitoring, intervention and rehabilitation as well^22^. Blood pressure (BP) monitoring can be carried out with several types of devices and sensors such as upper–arm electronic devices, multiparametric devices, smartphone applications paired with external BP sensors, cuffless smartphone–based BP monitors or wearable and tracking sensors^18^. A recent meta–analysis reported that, telemedicine based care of diabetic patients resulted in decreased level of glycosylated hemoglobin (HbA1c) after 12 months meaning a better glucose control, as well as reduced systolic BP just after 6 months^11^. Furthermore, the study showed improved self -management in rheumatoid arthritis patients^11^. Another study involved 49 patients with chronic diseases, and home–based telemonitoring of non–invasive BP, pulse oximetry, single channel electrocardiography (ECG), spirometry, body temperature, body weight, and blood glucose levels has been carried out by telemedicine nurses and doctors^22^. The vast majority of patients were satisfied with the telemedicine service (89.6%), and the participating healthcare providers (*n* = 9) responded that telemonitoring would potentially have a role in improving the overall quality of life of the patients and believed that their self–care and feeling of security has been also increased (89%)^22^.

A recent meta-synthesis has revealed that, patients are engaged towards remote health monitoring and telemedicine, also showed that, they prefer this methodology compared to regular clinical visits^23^.

To address the recurring challenge posed by the lack of digital competencies^24^ a possible solution could be a novel framework, which has been introduced to telemedicine care in Australia, in which a new role, the Clinical Care Coordinator has been established. They were responsible for patient monitoring as well as communication with GPs, specialists and nurses. Project Officers were also involved, in order to facilitate and manage the operational activities of the trial. The pilot project was successful, and drew attention to the point that the collaboration of different levels of care providers and specializations are essential in telemedicine-based treatment of chronic diseases^22^.

In some cases, like in telepsychiatry or telerehabilitation, virtual reality based solutions have been also investigated, and artificial intelligence can also support analysis and diagnostic steps^13,25^. However, it must be mentioned, that the availability of novel digital technology can further limit the possibility of complex care in rural and remote areas with lower income.

Based on previous international studies, it is highlighted that telemedicine based care is often restrained to a specific disease or discipline with limited technological approaches only including phone calls or telepresence solutions^3^. Consequently, these setup solely relies on self-reporting from patients therefore, the complex care of chronic diseases supported by digital technologies are still not fully explored, highlighting the significant need for further more complex studies of the field^3,24,26–29^. Also, in the majority of telemedicine-based workflows, as a drawback general practitioners (GP) and medical specialist do not take part in the monitoring or intervention in real time, they analyze the data, set up diagnosis and make interventions after data collection, which can be conducted by the patients or telemedicine nurses at home or medical sites^22,30^. In telemedicine supported home care programs, the self-data recording of health status, vital signs or symptoms performed by the patients is often not as reliable or adequate as data recorded in professional environment^31^. Besides logistical challenges, supply management and human resource issues, the lack of well–established workflows, population–level knowledge and regulatory background limit the applicability of telemedicine in these underserved regions^1,32,33^. A recent review highlighted that the majority of studies (75%) covering remote patient monitoring and the management of chronic health disease such as hypertension, diabetes mellitus and chronic obstructive pulmonary disease, and they mainly focus on patient adherence and the change of physiological parameters without patient management, which limited the effectivity of the telemedicine system^34^.

In Hungary, there is a notable variation in healthcare utilisation, reimbursement, and premature mortality rates across different geographical areas. In general, people living in segregated areas use health care services more frequently however, the amount of health care reimbursement paid for their care is significantly lower, suggesting lower quality of care^35^. To overcome this general social dilemma, the Hungarian Government with the help of well-established national or local non-government organizations launched a complex socioeconomic development program in 2019 in order to elevate the most disadvantaged 300 rural settlements of the country. The 300 settlements are listed as “Upcoming Settlements”, abbreviated as FeTe from the Hungarian expression (“Felzarkozo Telepules”). FeTe settlements are characterized by excessive disadvantages in housing, education and healthcare access, while having the highest rate of childbirth of the country. Thus, improving healthcare services in these locations is considered to be an essential part of the complex development efforts^36^.

The research groups of the Hungarian Charity Service of the Order of Malta, the University of Debrecen, and the University of Pécs are examining ways to support the digitalization of Hungarian healthcare services, with the aim of improving access to medical care in underserved regions. Based on the comprehensive analysis of the international literature, best practice guidelines and challenges associated with existing telemedicine systems, the research team developed a unique mobile, motorized healthcare unit. This unit is equipped with numerous medical tools including audiovisual platforms, telemedicine instruments, and POCT devices. The initiative aimed to test and validate this novel system by reaching out to patients in rural areas across the nation, therefore democratizing access to both general and specialist care for those experiencing shortages in healthcare services. The primary objective of the pilot study was to evaluate this innovative approach, which focuses on screening chronic diseases, providing remote specialist care by referrals on a public insurance covered basis, and validating a telemedicine ecosystem integrated into mobile healthcare units. To the best of our knowledge, this approach is unprecedented on a global scale, as it delivers specialist telemedicine care directly to underserved regions, with a nation-wide, unified system providing support and logistics. All significant steps and milestones are supervised and controlled by healthcare professionals, ensuring reliable data collection and processing. An initial cost-analysis has been carried out at the end of the pilot phase. Based on the hypothesis, the model can significantly improve the coverage and availability of telemedicine-based services in medical deserts, helping a wide population of patients at a national level.

## Materials and methods

### Study design

This study is a prospective observational pilot study evaluating the feasibility, cost-effectiveness, and impact of a mobile telemedicine system in underserved rural areas of Hungary. Rather than strictly following a predefined telemedicine or healthcare delivery framework, we adapted our approach to the specific challenges and needs of the target population to maximize effectiveness. Our methodology was shaped by practical considerations and real-world constraints, ensuring that the intervention was both feasible and responsive to the local healthcare landscape. The study was conducted over a six-month period (April–October 2023), assessing healthcare utilization, new diagnoses, and patient&doctor satisfaction.

The districts included in this study were selected from the 300 officially designated FeTe areas, which have been identified by the Hungarian Government and international organizations as the most socioeconomically disadvantaged regions. This externally validated classification ensured that the intervention targeted communities with the greatest documented need, while also mitigating potential bias in site selection. By focusing on these areas, we aimed to provide healthcare services where they were most urgently required, aligning with national and EU-supported socioeconomic development strategies.

#### Description of intervention

The mobile healthcare units were centrally operated from a single base in central Hungary, from where all vehicles departed each morning and returned the same day, covering an average of 500 km per trip. The target settlements were often difficult to access, with road conditions varying significantly between regions, impacting travel efficiency and scheduling.

A further challenge was ensuring stable internet connectivity for telemedicine consultations. The mobile network bandwidth varied across locations, influenced by both geographic factors and weather conditions, occasionally affecting data transmission quality.

Additionally, the diagnostic tools used in the intervention were originally designed for stationary use, making their daily transportation a logistical concern. Special precautions were required to prevent vibration, moisture exposure, and temperature fluctuations, ensuring that sensitive medical devices remained functional and accurate throughout the intervention.

Despite these challenges, operational adjustments, scheduling optimizations, and equipment protection protocols were implemented to maintain service quality and minimize disruptions.

### Description of the examined area

**Fig. 1:**
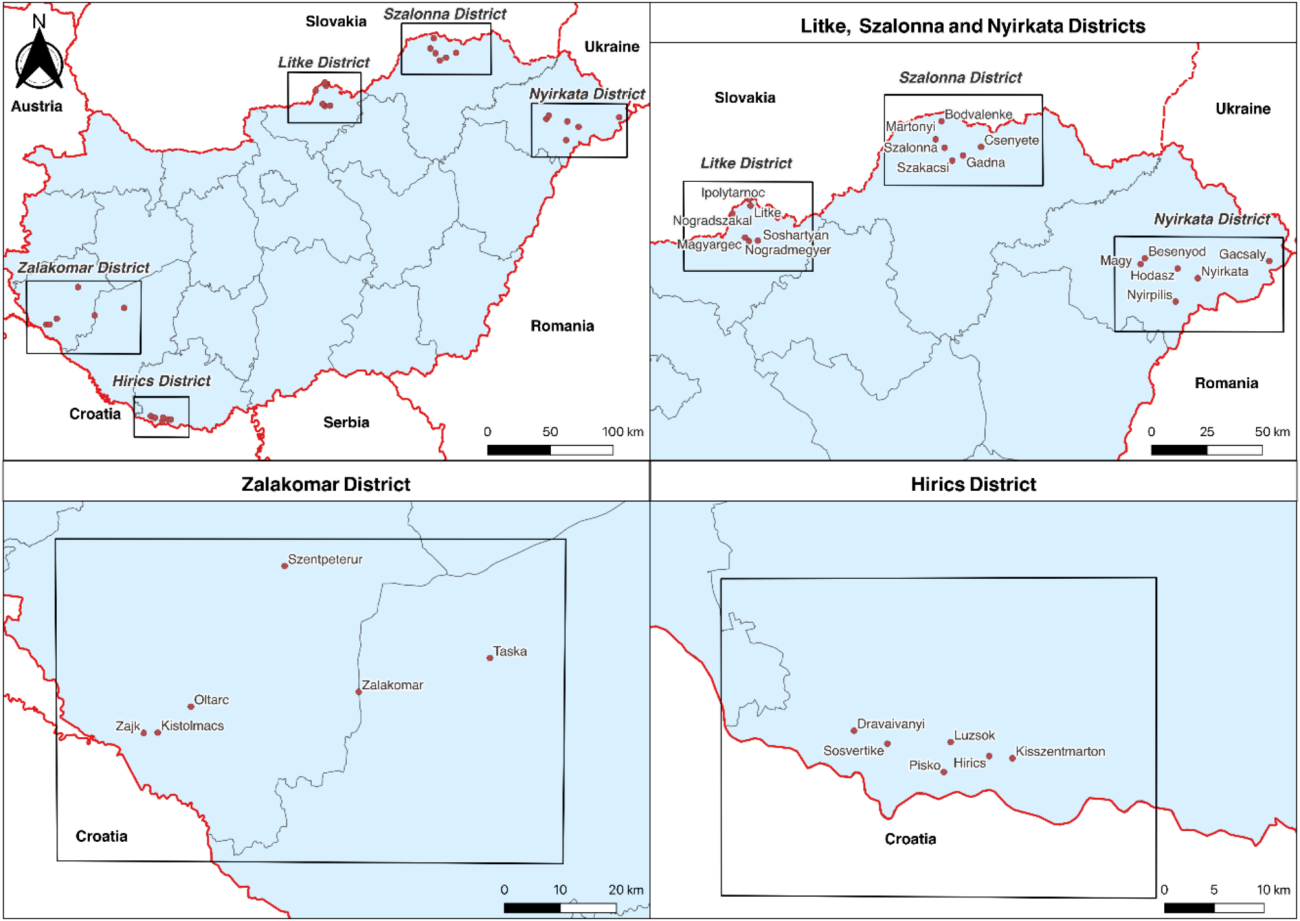
Districts involved in the programme. This figure shows the five rural Hungarian districts involved in the telemedicine programme: Zalakomar, Hirics, Litke, Szalonna, and Nyirkata. The map highlights these areas where mobile healthcare service centers (MHSCs) were deployed to provide medical services to underserved populations.

The introduction of our telemedicine system took place between April and October in 2023. The examined population came from 30 different locations, rural FeTe villages which constitute 5 districts, situated around 5 centers of telemedicine services. The 5 centers are the villages of Zalakomar, Hirics, Litke, Szalonna and Nyirkata (Fig. 1). Hereafter, the involved 5 districts and project together will be referred as “Programme”. The Programme served as the healthcare component of the socioeconomic development program in the FeTe settlements. To investigate the effectiveness of the nationwide telemedicine system, a preliminary analysis of population has been carried out.

Total population size of the five districts was 21,477 based on the 2022 Hungarian census. The control population was selected from other FeTe settlements based on their similar geographical location, socio–economic and demographic characteristics, but without Maltese Health Point (MHP) availability. MHP is a place that offers regular health service – telemedicine or traditional practice – and is operated and maintained by the Hungarian Charity Service of the Order of Malta (HCSOM). The complete list of 300 FeTe municipalities can be found in Annex 3 of Government Decision 1057/2021 (19.II.). In total, 171 control locations were selected with a respected population of 207,375 people in 2022 (*Supplementary Tables 1 - Control Locations*).

#### Population

To get a relevant and most up to date picture of the current medical state of the Programme area, demographic and socioeconomic factors were compared, to demonstrate the difficulties of the healthcare situation in the examined area. The demographic values (age distribution, crude live birth rate, crude mortality rate, educational level, proportion of the roma population) of the Programme were compared to the national averages, based on the 2022 Hungarian census database. For age distribution, according to the Hungarian census, three categories were applied namely, 0–20 years old; 20–60 years old and above 60 years old. The crude live birth rate, the crude mortality rate and the natural increase were analysed as well as the average life expectancy of the populations. To assess the complex socio-economic situation, the proportion of the roma population to the whole population just like the educational status of the residents in the Programme were examined.

#### Description of health care coverage

Of the 6342 general practices with designated service area in the country, 819 GPs (12,91%) are permanently vacated, meaning more than 6 months of absence of permanent medical staff. At the time of research, from the 32 GP practices in the Programme settlements, 15 (46.88%) practices were vacated and none of these practices were supplemented with permanent GP services. The national average for residents per doctor was 1,488.65 in 2022. The average in the Programme areas was considerably higher, namely 3,010 residents per doctor, indicating a severe shortage of healthcare providers. The standardized prevalence of GP-patient encounters in the Programme as a whole and in all individual districts were significantly higher than the national average (Table 1).

**Table 1:** Difference in GP–patient encounters. This table compares the Raw and standardized indicator values for GP–patient encounters between the programme areas, control areas, and National data. The values are expressed as percentages and include 95% confidence intervals (CI). Deviations from National and control data are highlighted, indicating where the programme significantly differs from these benchmarks.

Area	Raw indicator value for GPs (%)	Standardized indicator value in general practice districts (%) [95% CI]	Standardized indicator value deviation from the national data	Standardized indicator value deviation from control GP data
National	763,66	99,18 [99,15–99,20]	*N*.A.	*N*.A.
Control Area	980,32	128,70 [128,51–128,89]	significantly higher	*N*.A.
Programme – mean	966,00	126,06 [125,59–126,54]	significantly higher	significantly lower
Zalakomar	969,44	123,94 [122,99–124,90]	significantly higher	significantly lower
Hirics	1025,86	134,48 [133,16–135,81]	significantly higher	significantly higher
Litke	814,33	103,19 [102,18–104,21]	significantly higher	significantly lower
Szalonna	977,92	125,30 [123,99–126,62]	significantly higher	significantly lower
Nyirkata	1012,49	136,89 [136,04–137,76]	significantly higher	significantly higher

The proportion of people in the Programme who encountered outpatient specialist care with GP referrals (1.40%) was not significantly different from either the national average (1.68%) nor the control area (1.45%). The lowest rate of people attending outpatient specialist care with a GP referral was 1.09% and the highest value was 1,54% in the intervention area. All the districts were under the national thresholds, but only one (Litke) showed significantly lower referral rates than the national average (*Supplementary Tables 2 - Difference in outpatient specialist attendance*).

###  The mobile healthcare service centre

**Fig. 2:**
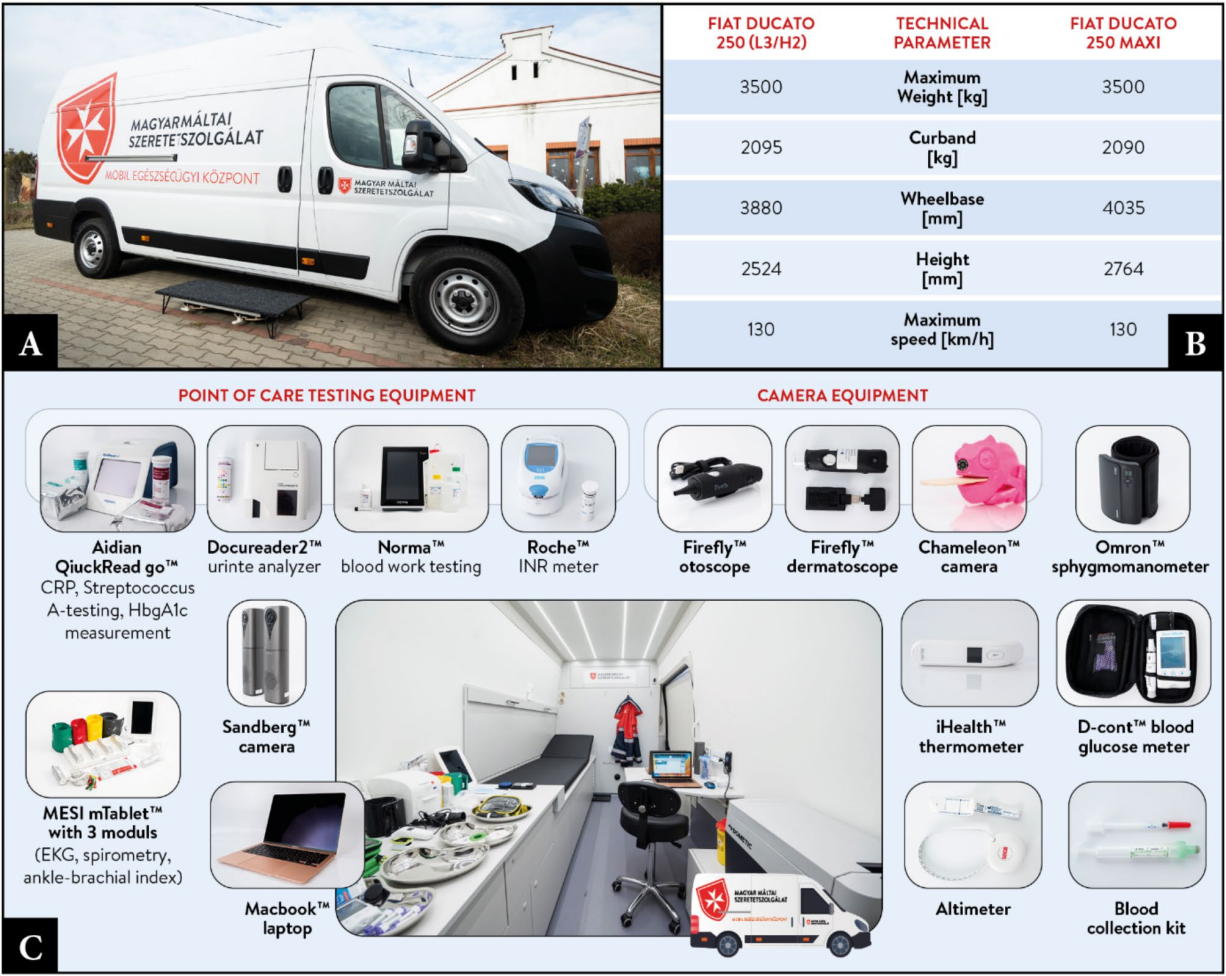
Mobile healthcare service center, developed and constructed in the programme. (**A**) Fiat Ducato 250 Max MHSC (**B**) main technical parameters of the vehicles (**C**) telemedicine and POCT devices applied on the MHSC.

In the Programme, healthcare services are delivered through specialized Mobile Healthcare Service Centers (MHSCs). These MHSCs are converted from Fiat Ducato Maxi 250™ or 250™ (L3/H2; Fiat™, Torino, Italy) vehicles (Fig. 2A ,B). Each vehicle was equipped with power aggregators, ensuring a stable power supply under all conditions, compatible with both 230 V and industrial power outlets (16/32 A). Due to the limited access to internet services in the settlements, two different internet providers were involved, namely Vodafone Group plc (Newbury, United Kingdom) and Telekom Hungary (Budapest, Hungary), with a Wi-Fi router (MikroTik™ HAP AC Lit; Riga, Latvia) optimized to connect to the strongest bandwidth vary from 4 Mb/s to 10 Mb/s on average. However, the available bandwidth varied widely across different locations. The lowest recorded download speed was 0.9 Mb/s, while the highest was 86.2 Mb/s. Upload speeds ranged from a minimum of 0.08 Mbit/s to a maximum of 66.3 Mb/s. The MHSCs are outfitted with telemedicine and POCT instruments to support screening and telespecialist consultations (Fig. 2C). This MHCSs enable to perform two levels of telemedical services which are general, basic medical support by GPs and specialized medical consultation. The general medical support provides an physical examination performed by a nurse and further telemedical support by GPs. The next level of care is the implementation of specialists with specialized medical knowledge in a given discipline, e.g. cardiologists, dermatologists, pulmonologists and endocrinologists assist patients in the context of telecare. Based on the results of initial physical examination, the nurse and the GP decide if a specialist should be involved to the care. The visual assessments were conducted by a Firefly™ (Firefly Global, Belmont, Massachusetts, United States) camera system, which includes an otoscope, dermatoscope, and general camera. As point-of-care tests, an Aidan QuickRead Go™ (Aidan, Espoo, Finland) has been installed for rapid c-reactive protein (CRP), Streptococcus-A, and HgbA1C measurements; a Docureader 2™ (77 Elektronika Ltd., Budapest, Hungary) for urine examinations (glucose, ketones, specific gravity, blood, pH, protein, nitrites, bilirubin, urobilinogen, leukocytes); Roche CoaguCheck™ (Roche, Basel, Switzerland) for prothrombin time test (INR) and a Norma™ for blood testing (complete blood count, hemoglobin, hematocrit, white blood cell count, platelet count). MESI™ (MESI Ltd., Ljubljana, Slovenia) system was implemented for ECG testing, spirometry, and ankle-brachial index measurements. Body temperature was measured with an iHealth™ (Sunnyvale, California, United States) thermometer, and fast glucose level screening was conducted by a D-cont™ (77 Elektronika Ltd., Budapest, Hungary) blood sugar testing device. For screening of heart and lung sounds, a digital phonendoscope was used: EKO Duo™ (Emeryville, California, United States) and Thinkabs™ (Centennial, Colorado, United States). The audiovisual connection between the patient, healthcare worker, and telespecialist doctors was supported by a MacBook Pro™ (Apple Inc., Cupertino, California, United States) and a Sandberg™ (Sandberg, Birkerød, Denmark) camera. Patient management were supported by a web-based software solution, NetDoktor™ (Dericom Ltd; Budapest, Hungary). (Fig. 2C) Overall, 12 MHSCs were involved in the Programme.

#### Telemedicine intervention

The telemedicine intervention provided a wide range of healthcare services tailored to the needs of rural populations. Primary prevention was facilitated through community-based health education events known as “Health Forums,” which were regularly held in all serviced settlements. Secondary prevention focused on cardiometabolic screening programs, enabling early detection of chronic conditions. In addition to preventive care, the system offered acute care for a limited range of indications, although certain conditions, such as abdominal complaints, posed challenges due to the limitations of remote diagnostics. Long-term follow-up was available for chronic disease management, ensuring continuity of care. The intervention also optimized patient pathways, facilitating diagnostic coordination and referral efficiency. Furthermore, distinct ultrasound services were integrated, enhancing diagnostic capabilities. These services were available to all patients, regardless of age, from infancy to elderly individuals.

The system relied on a specialized medical software platform designed specifically for Hungarian healthcare providers. This was supplemented by videoconferencing software for teleconsultations with specialists and proprietary diagnostic software (e.g., MESI for ECG, spirometry, and ankle-brachial index measurements). Database management and administrative tasks were supported by Microsoft products, ensuring seamless data handling.

Healthcare professionals involved in the intervention underwent structured training programs. Nurses participated in regular training sessions, with new employees undergoing a three-month training period before full integration to the program. Additionally, a dedicated training day was scheduled at least once per month for continuous skill development. Medical doctors were also trained to use telemedicine diagnostic tools before joining the team and were required to attend webinars at least twice a month to stay updated on best practices.

### Cost analysis

The publicly funded single insurer healthcare system of Hungary provided the opportunity to use publicly available databases to assess the per capita spending in primary care in various settings. As the publicly funded Hungarian primary healthcare is financed using a flat rate mostly based on the size and age constitution of the practice population, knowing the size and type of the practices, the typical spending for the various practice types could be determined. The payments for all healthcare providers are published monthly by the National Health Insurance Fund (Nemzeti Egeszsegbiztositasi Alapkezelo, NEAK). In a separate database, the NEAK also publishes monthly status of all primary care practices including location, population size, type (urban adult, urban pediatric or rural mixed practices) and status (vacant or occupied). Dor the calculations, these two databases were matched. In order to maintain the consistency of the calculations, financial data from providers operating multiple primary care practices with different status had to be excluded, as the financial data were published at the provider and not at the individual practice level (data for 173 of the total 6342 practices were excluded for this reason). If a single provider operated multiple practices with the same status, single practice spending was assumed as the average per praxis spending of the provider. Per capita monthly spending in primary care then was calculated for every practice in the exam group using the individual practices’ population data as a denominator.

Since initial data was given in HUF, the calculations were made in HUF as well. The costs were in HUF, and to reflect this, the calculations were also made in HUF. However, to ease the understanding, the final results are presented in US dollar terms, converted on mid-rate (published by Hungarian National Bank) and 350.20 was used for the USD/HUF currency pair.

###  User feedback questionnaire

To evaluate the experience and satisfaction of patients and doctors with the telemedicine system of the Programme, a structured user feedback questionnaire was created by the research team. The questionnaire was designed to capture various aspects of the user experience, and it contained closed–ended questions, which utilized Likert scales to measure satisfaction, likelihood of returning, and likelihood of recommending the service to others.

The questionnaire was distributed to patients following their telemedicine consultation, and responses were collected either electronically or via paper forms depending on patient preference or accessibility. A total of 206 out of 1,889 patients completed the questionnaire. (The translated patient questionnaire can be found in *Supplementary Material 1 - Patient feedback questionnaire*) In parallel, a separate questionnaire was designed for the healthcare providers who participated in the telemedicine intervention. This questionnaire aimed to assess their experiences with the telemedicine system, the perceived quality of care delivered through this medium, and their overall satisfaction with the telemedicine workflow. The doctor questionnaire included questions on the frequency of use, the perceived quality of telemedicine care compared to in–person care, and their willingness to continue using telemedicine in the future. A total of 17 out of 24 doctors filled out the questionnaire. (The translated doctor questionnaire can be found in *Supplementary Material 2 - Doctor feedback questionnaire*)

### Data and statistical analysis

The demographic and socio–economic data were obtained from the Central Statistical Office census data for 2022, including age, gender, educational attainment, income level, and economic activity, while the health data was provided by NEAK. A register of all patients who used a healthcare service provided by the intervention program was created, including sociodemographic data, economic activity, participation in public health screenings, and the purpose of attendance. Patients without a social security number in the register were excluded from the analysis. Statistical analyses were conducted using Jamovi 2.3.28 software. For descriptive statistics, we calculated the number of cases (n), proportions (%), and corresponding 95% confidence intervals for the indicator values. Differences between the program and national values/ratios were assessed using the Mann–Whitney U test due to non–normal distribution and lack of homogeneity in the sample, as indicated by pre–testing (Levene’s test). The raw indicator values were calculated as percentages (%). After adjusting for age, sex, and eligibility for public health care, we calculated the standardized indicator values for the baseline period, December 2021. Standardized prevalence ratios were used to characterize the indicators, accounting for the effects of patient age, sex, and eligibility for public health care as proxy variables for socioeconomic status. Additionally, we determined the 95% confidence intervals of the standardized prevalence coefficients, which were used to assess the variability of quantifiable indicator values in GP districts in the program area compared to those in the national or control areas. Mortality tables were used to estimate life expectancy at birth. Mortality and life expectancy data were derived from NEAK’s aggregated mortality tables, using the Chiang method for settlement-level life expectancy calculations^37^. When carrying out the financial analysis the average per capita spending with standard deviation was calculated for the subpopulations of practices of various types and vacancy status using Microsoft Excel. Data are presented as mean ± SEM (Standard Error of the Mean).

The analysis was conducted using Jamovi 2.3.28.

Descriptive statistics included case counts (n), proportions (%), and 95% confidence intervals.

Regarding subgroup analyses, stratifications based on age, gender, and district-level differences were examined in key outcome measures. These analyses provided additional insights into variations in healthcare utilization and service accessibility within the intervention areas.

Regarding data collection challenges, missing data and technological limitations in certain rural areas were mitigated through standardized data collection procedures and validation mechanisms. In cases where healthcare utilization data were incomplete, cross-referencing with census-based population statistics allowed for improved data accuracy.

###  Data handling and ethical considerations

The study was approved by the Hungarian Medical Research Council (ETT–TUKEB; BM/9240– 3 /2023). All the patient data were anonymized before analysis, and written consent has been obtained by the participants. All methods were carried out in accordance with relevant regulations and the study was conducted in accordance with the Helsinki Declaration.

The ethical principles and data privacy measures applied in this telemedicine intervention align with standard medical practice and adhere to national and EU data protection regulations, including GDPR (General Data Protection Regulation). All patient data, including electronic health records and telemedicine consultation details, are encrypted and securely stored to prevent unauthorized access. The videoconferencing communication channel used for remote consultations is end-to-end encrypted, ensuring the confidentiality of patient-doctor interactions.

Additionally, all informed consent statements are signed by both the patient and the attending nurse, and they are systematically collected and stored to ensure compliance with data protection regulations.

##  Results

The aim of the pilot research project was to assess the health situation in the intervention area and to get a more accurate picture of the health status, demographic and socio–economic situation of the people living in the Programme area and to narrow the gap in health care provision through various services. Interventions include basic screening tests to diagnose patients who have been previously undiagnosed. In addition, the activity included care of acute cases, care of chronic patients and assistance with patient journey planning. The latter consisted of both local telemedicine based care and referrals to hospital-based specialist care.

### Characteristics of the examined population

The demographic analysis revealed distinct differences between the Programme areas and national averages. The age distribution showed that the proportion of people aged 0–20 years in the Programme was 28.59%, compared to the national average of 19.57% (*p* < 0.001, *r* = 0.732). Similarly, the proportion of active adults aged 20 to 60 years was 51.84% in the Programme compared to the 53.93% national value (*p* = 0.293, *r* = 0.111). Conversely, the elderly population (60 years and above) was lower in the Programme areas at 19.47%, vs. national rate of 26.50% (*p* < 0.001, *r* = 0.665). The national crude live birth rate in 2021 was 9.6‰ while in the Programme it was 16.12‰. In 2021 the value of crude mortality rate in Hungary was 15.93‰, compared to the 12.01‰ in the Programme. In 2022, the average natural decrease per 1,000 inhabitants was 6.4 nationally. Conversely, the Programme population had an average increase of 4.11, with a minimum value of -3.05 and a maximum 18.87. This shows a positive natural increase in the intervention area in contrast with the national natural decrease of the population (*p* < 0.001, *r* = 0.392). Average life expectancy was lower in all age groups (3, 18, 25, 45, and 65) in the Programme areas compared to national indicators, and this gap persisted across all examined age groups, but the differences were not significant.

**Fig. 3:**
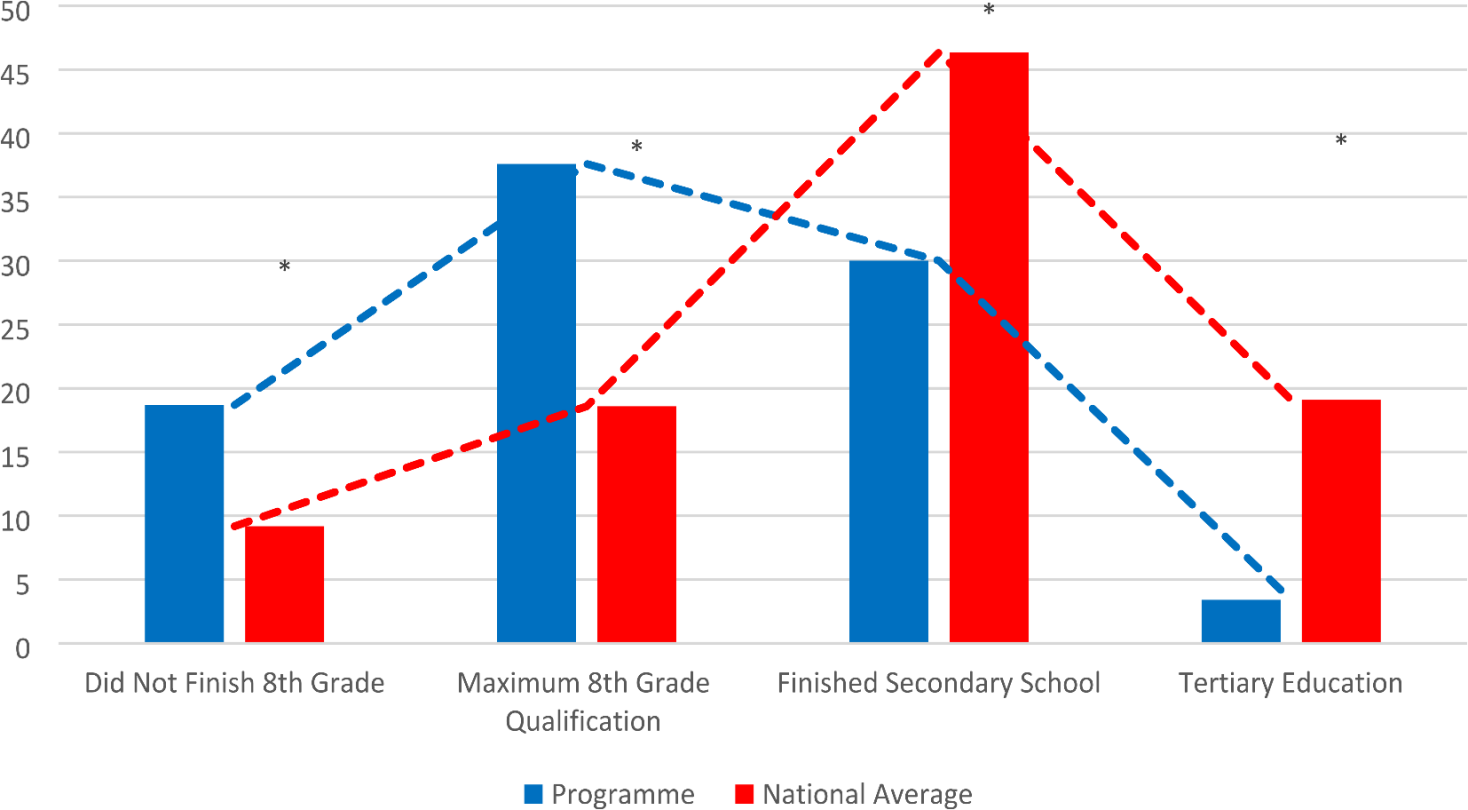
Educational status. educational status in the programme areas compared to national averages. This figure illustrates the differences in educational attainment between the Programme areas and the national averages in Hungary. The data highlight disparities across four educational categories (namely: Did not finish 8th grade, Maximum 8th grade qualification, Finished secondary school, and Tertiary education), showing a higher proportion of individuals with lower educational levels in the Programme areas compared to national figures.

The socio-economic analysis showed that the average proportion of the roma population in the Programme intervention area was 20.50% (minimum 3.06%, maximum 90.46%), compared to the 2022 national average of 2.19% (*p* < 0.001, *r* = 0.833). In the Programme 18.69% of people didn’t finish the eighth grade of primary school, compared with 9.18% national value (*p* < 0.001, *r* = 0.855). 37.60% of the people in the intervention population had maximum 8th grade qualification, in contrast with 18.59% in the country as a whole (*p* < 0.001, *r* = 0.878). While 46.34% of Hungarians finished secondary school, in the Programme this proportion was 30.01% (*p* = 0.001, *r* = 0.823). People with tertiary level education are represented in the Programme with 3.41% while nationally this value is 19.09% (*p* < 0.001, *r* = 0.782). (Fig. 3).

### Services provided during the intervention

**Fig. 4:**
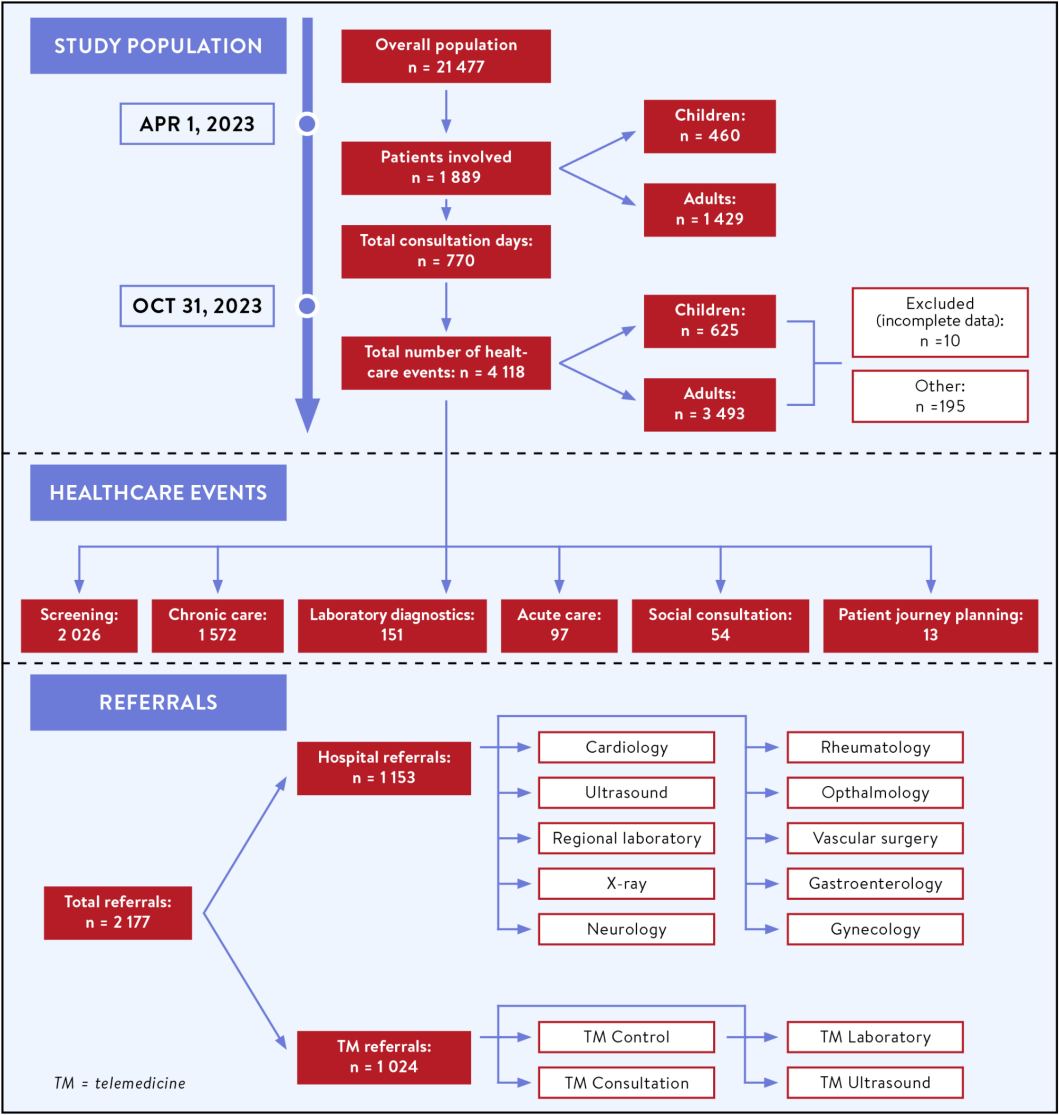
Healthcare Services provided in the Programme. The figure shows the types of healthcare services delivered during the intervention. The services are divided into categories such as screenings, chronic care, acute care, social consultation, referrals, laboratory diagnostics, and patient journey planning, highlighting the frequency of each type of service provided across the population.

In the study, conducted between April and October 2023, the total population within the districts involved in the Programme was 21,477. During this period, 770 consultation days were held, reaching a total of 1,889 individuals (8.80% of the overall population). Of these, 1,429 were adults and 460 were children, each attending at least one consultation session. The total number of physicians delivered telemedicine care events was 4,118. 3493 care events were reported related to adults and 625 related to children, in10 cases the age specific data was missing and in 195 cases were categorized as “other” because the fillings were uninterpretable. The average number of care events was 2.44 for each adult and 1.36 for each child, respectively. In total 2,026 screenings, 1,572 chronic care, 151 laboratory diagnostics, 97 acute care, 54 social consultations and 13 patient journey planning were carried out in the examined period. The mean (± SD) time per care event was 32.13 min (± 15.18) for adults and 33.41 min (± 15.35) for children, for a total of 32.27 min (± 15.20). We conducted 2,177 referrals, from which 1,153 were regular hospital referrals (Cardiology, Ultrasound, Regional laboratory, X-ray, Neurology, Rheumatology, Ophthalmology, Vascular surgery, Gastroenterology, Gynecology) and 1,024 were telemedicine referrals (TM Control, TM Consultation, TM Laboratory, TM Ultrasound). (Fig. 4)

###  Use of telemedicine devices and instruments

**Fig. 5:**
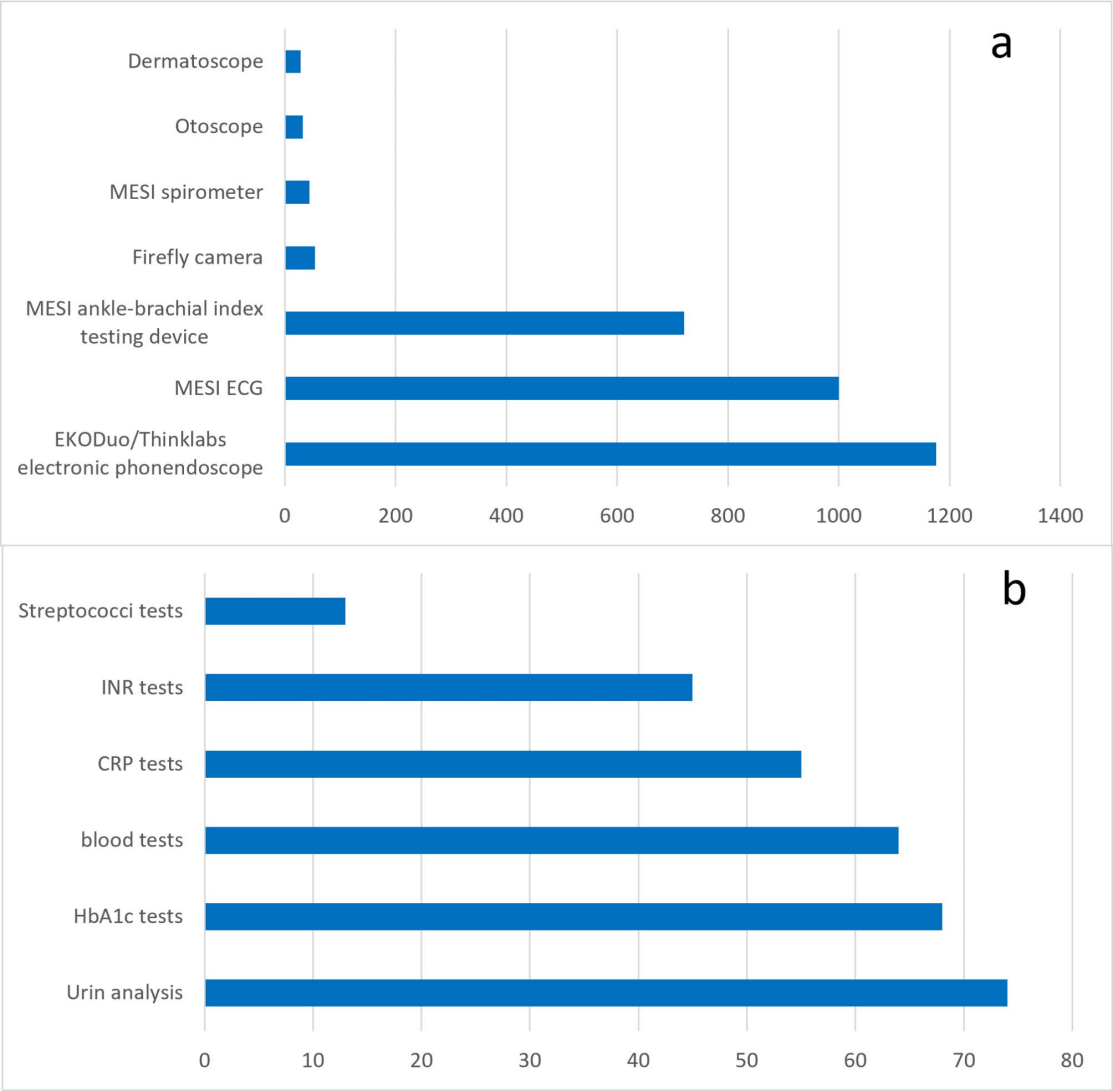
Utilization of Telemedicine Tools and POCTs in the Programme. (**A**) The use of various telemedicine tools throughout the Programme, including electronic stethoscopes, ECG devices, ankle-brachial index measurement tools, and otoscopes. The frequency of usage varied, with stethoscopes and ECGs being the most commonly applied devices. (**B**) The distribution of POCTs conducted during the intervention, including urine analysis, HbA1c, blood tests, CRP, INR, and Streptococci tests. POCTs were used in a smaller proportion of cases compared to telemedicine tools but played a key role in diagnostic support.

The research team gathered patient encounters on monthly basis. In terms of device usage, every patient counted maximum once per month regardless the number of visits and repeated examinations. This data aggregation resulted in one person being counted once in a given month, but more than once over the whole intervention period if the patient returned one or more months later. Based on this data collection method 2,550 people were served during the 6 month period. From the aggregated 2,550 cases 83.29% were adults and 16.71% were children. Blood sampling was the most frequently performed care event, as it was executed 935 times, and with that 36.67% of the visitors were involved. 21 patients (0.82% of the attendants) had an emergency condition that justified a call for an ambulance during the half year period. Regarding the telemedicine tools and tests, the most frequently used devices were EKODuo/Thinklabs™ electronic phonendoscope (*n* = 1176; 46.12%). The second most popular tool was the MESI™ ECG electrocardiograph (*n* = 1000; 39.22%), which was followed by the MESI ankle-brachial index testing device for measuring peripheral blood pressure (*n* = 721; 33.95%) and the Firefly™ camera otoscope (*n* = 55; 2.16%). MESI™ spirometer was used in 46 cases (1.80%), regular otoscope in 33 cases (1.29%) and a dermatoscope in 29 cases (1.14%). (Fig. 5A)

From the 2550 cases in total, 319 (12.51%) occasions involved the application of POCTs. The most frequent laboratory test was urine analysis (n=74). 68 HbA1c tests were used, 64 blood tests, 55 CRP tests, 45 INR tests and 13 Streptococci tests. (Fig 5B) Acute care indicated the use of burning gel in 45 (1.76%) cases, oxygen in 3 (0.12%), crystalloid (Isolyte) in 1 (0.04%) occasion. Telemedical check-ups were performed 682 times (31.33%), telemedical consultations 32 times (1.47%) and telemedical laboratory tests 148 times (6.80%).

###  Outcomes of healthcare services

1,612 (on 46.15% of the 3,493 adult cares) adult screening events were registered during the intervention. They were screened for cardiovascular diseases and diabetes mellitus. During the adult patient care and screening events 1,430 patient’s blood pressure and blood glucose level were checked, which resulted in 105 (5.56% of the examined patients) new hypertension and 26 (1.38% of the examined patients) new diabetic cases.

To gather accurate information about the residents’ health coverage, the participation status of Programme participants regarding to recommended preventive screening for malignant diseases were examined as well via interview questions. Based on the results, 19.55% (*n* = 158) of women aged 25–65 years participated in cervical cancer screening activities in the 3 years prior to the interview among Programme participants. 13.17% (*n* = 59) of women aged 45–65 in the breast screening target group had a mammogram in the previous 2 years. 1.78% of all adults aged 50–70 years (*n* = 10) in the colorectal cancer screening target group had a screening test (colonoscopy or fecal occult blood testing) in the last 2 years.

During the first check-ups particular attention was given to common and important risk factors affecting health, such as obesity, alcohol consumption and smoking. 1077 Body Mass Index (BMI) and 898 abdominal circumference measurements were carried out during the 6 months of the Programme. 701 (65.08%) patients had 24.99 kg/m^2^ or higher BMI and 589 (65.59%) had higher abdominal circumference then the threshold normal value (88 cm for women and 102 cm for men). Detailed data by districts is presented in *Supplementary Tables 3 - BMI in the examined population* and *Supplementary Tables 4 - Abdominal circumference in the examined population*. The national ratio of people with more than 24.99 kg/m^2^ BMI was 58,2%, while in the Programme, it was 65.09%.

From the interviewed 1,271 patients 624 smokers were identified (*Supplementary Tables 5 - Regular smokers in the examined population*). In the Programme 49.10% of the asked people were smokers compared to the 27.2% of the 2019 national value. From the interviewed 1,210 patients 475 regular drinkers were identified (*Supplementary Tables 6 - Regular alcohol consumers in the examined population*). In the Programme 39.26% of the asked people drink alcohol on a regular basis in comparison with the 25.4% of the 2019 national value.

**Fig. 6:**
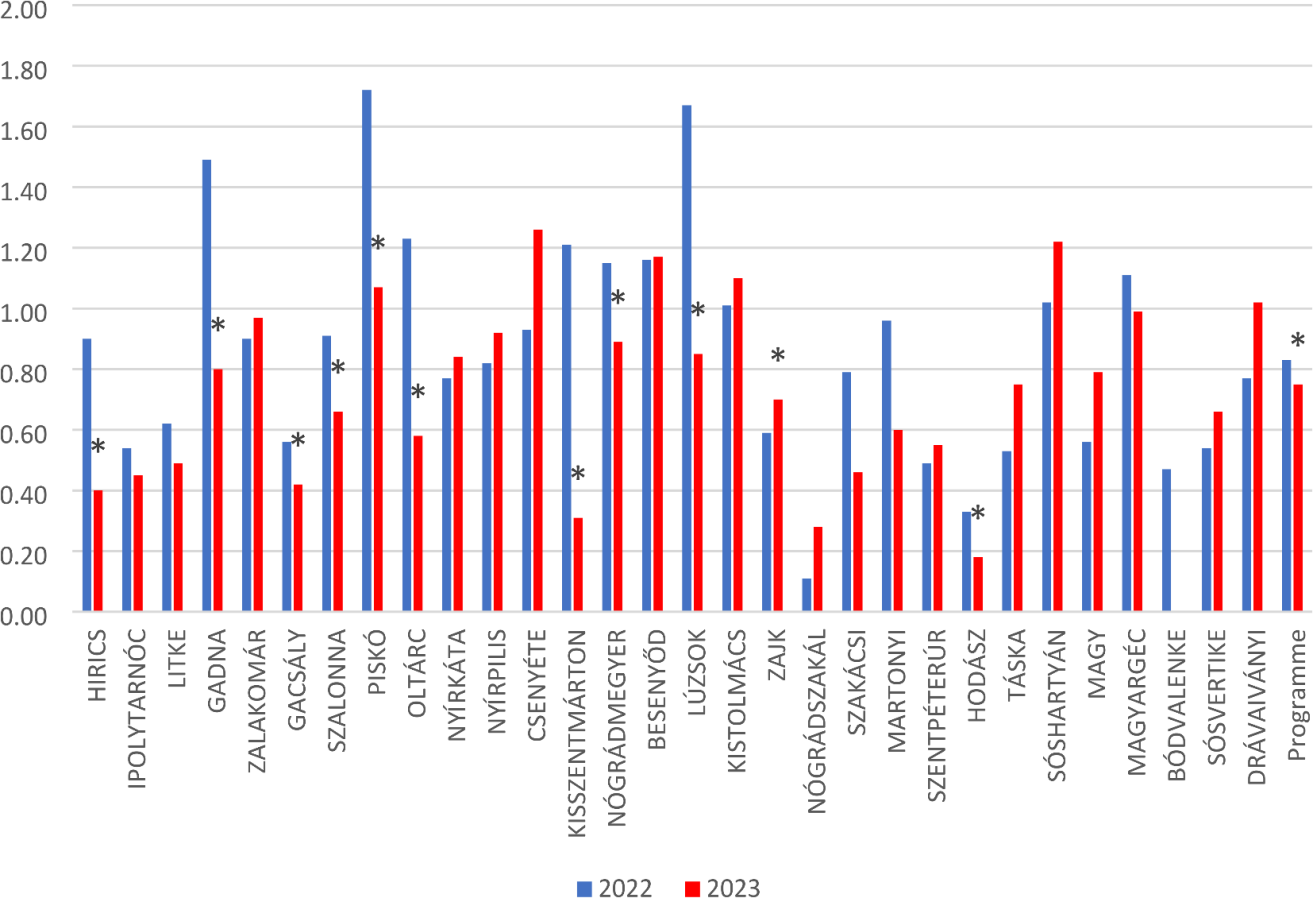
Changes in relative referral rates in the intervention area. This figure illustrates the differences in referral rates between the intervention and control areas before and after the implementation of the Programme. Significant reductions in referral rates are marked with an asterisk, highlighting the potential impact of the intervention on decreasing the need for external healthcare referrals.

During the intervention period, 987 patients received a total of 2,177 referrals for further investigation. 1024 were referred to telemedicine units with specialist consultation, and 1,153 to healthcare institutions such as clinical departments or regional hospitals. The 1,024 telemedicine referrals consisted of 682 control examinations, 32 consultations, 148 laboratory tests and 162 ultrasound examinations. The 10 most frequent referrals to traditional healthcare units were to cardiology (*n* = 168; 14.57%), ultrasound (*n* = 116; 10.06%), regional laboratory (*n* = 98; 8.50%), X-ray (*n* = 97; 8.41%), neurology (*n* = 69; 5.98%), rheumatology (*n* = 67; 5.81%) ophthalmology (*n* = 51; 4.42%), vascular surgery (*n* = 43; 3.73%), gastroenterology (*n* = 40; 3.47%) and gynaecology (*n* = 37; 3.21%). (Fig. 4) Based on the control area the expected referral count was 3664, but only 2756 were registered by NEAK in 2023. The 10 most frequent referrals from the control areas were regional laboratory (*n* = 1290; 49.81%) X-ray (*n* = 188; 6.82%), pulmonology (*n* = 186; 6.75%), ultrasound (*n* = 155; 5.62%), rheumatology (*n* = 124; 4.50%), internal medicine (*n* = 127; 4.61%), neurology (*n* = 101; 3.66%), cardiology (*n* = 73; 2.65%), orthopedic (*n* = 73; 2.65%) and surgery (*n* = 61; 2.21%) referrals. In terms of total referrals, the examined area typically had a lower referral rate in the year before the Programme than the control area in general (Relative Referral 2022: 0.83 [0.80;0.86]), which decreased further in 2023 (RR2023: 0.75 [0.72;0.77]). Overall, a 10% significant decrease in relative referral frequency was detected (RR change: 0.9 [0.86;0.95]) We found significant decrease in the relative referral rate 11 out of the 30 villages, namely Hirics, Gadna, Gacsaly, Szalonna, Pisko, Oltarc, Kisszentmarton, Nogradmegyer, Luzsok, Zak, Hodasz, (Fig. 6).

### User feedback

206 feedback was collected from the Programme patients. 146 women (70.87%) and 60 men (29.13%) filled the questionnaire.

In response to the question “Did you have any unanswered questions after the examination?” 206 valid answers were received. 3.4% of respondents (*n* = 7) answered ‘Unfortunately not’, 5.34% (*n* = 11) answered ‘Maybe.’ and 91.26% (*n* = 188) ‘Not left. All my questions were answered’.

The question “How did it feel to interact with your doctor via video online?” was answered by 122 patients (52.22% of the responses). 7.37% (*n* = 9) of the patients found it acceptable, 0.81% (*n* = 10) found it strange, 28.69% (*n* = 35) said it was new and exciting, 0.81% (*n* = 10) said it was uncomfortable and 62.29% (*n* = 76) found it natural.

A four-point Likert scale was used to answer the items “Satisfied”, “Would come back”, “Would recommend” with the following options: “Absolutely/very definitely”, “Probably/most probably yes”, “Rather no”, “Definitely/certainly no”. 96.11% (*n* = 198) of respondents were completely satisfied with the care they received, while 3.88% (*n* = 8) answered “More likely yes”. 95.14% (*n* = 196) would definitely come back and 4.85% (*n* = 10) would probably come back. 96.11% (*n* = 198) would definitely recommend the service to others and the remaining 3.88% (*n* = 8) would probably recommend the service. Nobody chose from the “Rather no” or the “Definitely/certainly no” items on the scale.

Of the 27 doctors who participated in the Programme, 17 (62.96%) completed our online questionnaire about their experience of the telemedicine system. Doctors used the system on different extent, namely: 5 (29.41%) doctors interacted with less than 20 patients, 4 (23.53%) of the 17 have tended 20 to 100 patients and 8 (47.06%) of the respondents dealt with more than 100 patients.

When the professional point of view was explored based on the response regarding how would they rate the Programme compared to other job, 8 (47.06%) doctors answered that it provides a much higher quality, 4 (23.53%) said that it is about the same, 1 (5.88%) thought that the quality is somewhat lower and 4 (23.53%) doctors said that it provides a much lower quality. Nevertheless 15 (88.24%) doctors would recommend the telemedicine job for other colleagues for sure, and 2 (11.76%) maybe. Furthermore 12 (70.59%) respondents want to continue the work in the Programme for sure and 5 (29.41%) of the 17 doctors would rather continue it.

The doctors were also asked after each patient meeting about their thoughts if they were allowed to carry through the care event in person, how high quality could they achieve compared to the telecare event? Out of the 1,572 care events analyzed, caregivers reported that in 77.99% of the cases (*n* = 1,226), they were able to achieve the same quality of care as they would in person. In 16.03% of the cases (*n* = 252), they believed they could provide even better care, while in 5.98% of the cases (*n* = 94), they felt they could have delivered a higher quality of care in person.

### Financial analysis

The population of FeTe settlements is underserved medically in many areas, including primary care. In the Program settlements nearly half of the GP practices are vacant (15/32), leaving 47.87% of the Program population (10,280 of 21,477) without a permanent GP. If the corresponding GP practice is vacant, the municipality is by law required to fill in 50% of the official visiting hours of the practice by locum doctors, which still results in reduced healthcare access for the population. In addition, NEAK offers reduced financing to these vacant practices leading to a more complex and ongoing issue. All the FeTe settlements in the program have rural, mixed primary care practices. The national average per capita primary healthcare spending for mixed rural practices in the country (*n* = 1097) with a permanent GP is 7.51 ± 0.08 USD per capita per month. Assuming an ideal situation, where all GP practices are filled in the Programme area, the cost would be 161,510 ± 1,730 USD/month for the 21,477 residents. If we calculate with the national non-rural GP coverage (91.70%) the cost would be 155,190 ± 1,760 USD/month per capita. In comparison, the actual primary healthcare spending according to the NEAK database is just 95,140 USD/month resulting in 60,050 to 66,370 USD monthly underfunding for the Program population compared to the realistic or ideal scenarios.

Aiming to fill in the vacancies, NEAK also runs a stipendium program, offering up to 57,110 USD upfront single payments to doctors who would sign a minimum 6-year contract as permanent GPs in practices that have been vacant for more than 12 months. All 15 vacant practices of the Program would be available for such funding, which would mean 856,650 USD extra to recover the primary healthcare services in the Programme area.

Regarding to the telemedicine system it costs around 242,720 USD to 257,000 USD to operate 12 MHSCs on monthly basis, including the salaries of staff (doctors, nurses, telespecialists and administrators), the cost of tests (laboratory, equipment, etc.), the cost of running and maintaining vehicles too, leading to an average monthly upkeep cost of 20,226 USD to 21,417 USD per vehicle. (Table 2)

**Table 2:** Financial overview of the healthcare spendings in the programme. This table provides an overview of the financial aspects of the programme. including the number of vacant GP practices, population affected by the vacancies, and monthly per capita healthcare spending. It compares actual spending in the programme.area with ideal and National averages. Additionally, the table lists the costs associated with running mobile healthcare service centers and the estimated budget shortfall in the programme.region. Values are presented in USD with standard deviations (± SD) where applicable).

Category	Value
Total population of FeTe settlements	21,477
Vacant GP practices (out of 32)	15
Population without permanent GP	10,280 (47.87%)
National average per capita spending	7.51 ± 0.08 USD/month
Ideal scenario cost for all practices	161,510 ± 1,730 USD/month
National non***–*** rural GP coverage cost	155,190 ± 1,760 USD/month
Actual healthcare spending	95,140 USD/month
Monthly underfunding	60,050 to 66,370 USD
Average cost of 12 Mobile Healthcare Service Centers	242,720 USD to 257,000 USD
Average cost of a single Mobile Healthcare Service Center	20,226 USD to 21,417 USD

## Discussion

Telemedicine is a continuously evolving, special form of healthcare services, which merge both technological and methodological advancements in medicine. In the last two decades, the rapid development of ‘Internet of Things’ solutions, telemedicine instruments and the increasing availability of broadband internet paved the way towards innovative approaches, enhancing the opportunity to seek medical care in rural areas.

In the presented study, a nationwide pilot action has been implemented, with a new and unique form of telemedicine-based healthcare services in remote areas of Hungary. Overall, 12 Mobile Healthcare Service Centers covered 5 districts, with a total population of 21,477 inhabitants. Interestingly, only a limited number of studies report vehicle mounted telemedicine systems, and previous projects targeted acute care, equipping digital instruments to ambulance cars also, only preliminary studies investigated the potential use of mobile health clinics, without the detailed description of technical approach, introduction of referrals or patient outcomes^5,6,38^.

Within the 6 months of intervention, 1,889 patients with public insurance were reached and examined in MHSCs, within 770 consultation days. The MHSCs are equipped with telemedicine devices, POCT instruments and IT solutions provided audiovisual connection with medical specialists. The developed telemedicine ecosystem aimed to screen chronic diseases and facilitate referrals to regional hospitals or clinical departments, while providing specialized medical care from distance.

It is demonstrated that the region involved in the Programme, has a population characterized with significantly lower socioeconomic status, compared to national averages. Based on the results, 105 new hypertension and 26 new diabetes cases were diagnosed and treated / referred / guided with instructions, enabling them to start the treatment and follow-up leading to health benefits in a long term. The number of hidden or undiagnosed cases can not be determined with certainty, but it is assumed, that potentially hidden cases were revealed, and this question needs thorough examination in future studies. On follow-ups and chronic care events crucial health parameters of hypertonic, diabetic and obese patients were measured in order to assess the health benefits of the Programme. However, presumably due to the short duration of the study, a significant change was not observed in the health parameters of the patients. It is believed that with further reciprocations and continuation of the intervention, the health gain will be relayed and further improved.

Also, the health behaviors connected to main lifestyle risk factors have been assessed, like smoking, alcohol consumption and obesity. The results indicated significantly higher prevalence of these risk factors in the Programme areas compared to national averages, which underline the importance of telemedicine-based screening in remote areas, as secondary prevention. Follow-ups were arranged, in the hope that it could make a positive impact on the lifestyle and health of the involved patients. In the future, further measurements are planned to measure the impact of the mobile telemedicine ecosystem.

As one of the main outcomes of the project, the referral activity of the Programme was similar to conventional GP referrals, in terms of case numbers and the distribution of specializations. 987 patients received referrals, which is 51.97% of the involved population. This indicates the potential of our telemedicine method, nominating the system to be an effective alternative for standard healthcare services, therefore in the future it can effectively support primary care of the underdeveloped areas of the country. Furthermore, there was a significant decrease in the relative referral rate in the intervention area compared to the control area, which represents the effectiveness of the telemedical intervention in case of relieving the healthcare system, which has a potential positive impact on national-level healthcare expenses. The feedback from the doctors and patients also indicates the success of the telemedicine system. According to the results, a high level of patient satisfaction was observed, with over 96% of respondents being completely satisfied with the care received and would recommend the service to others. Additionally, besides the patients, most of the practicing doctors expressed positive views about the quality of care provided through telemedicine and their willingness to continue using the system in the future. Based on the results obtained, we believe that the developed and applied telemedicine ecosystem with specialized remote care is suitable to improve the situation in underdeveloped areas and reduce the identified gaps in health care, as previous studies indicate in other countries^34^.

From an international perspective, mobile telemedicine programs are widely used, for example, in Australia and the United States, particularly in remote areas with limited healthcare access^5,22^. These systems help reduce travel burdens, improve chronic disease monitoring, and lower hospitalization rates. Further comparisons with similar telemedicine programs could help contextualize the effectiveness of this intervention. While our findings suggest reduced referral rates, high patient satisfaction, and effective specialist consultations, long-term studies are needed to assess chronic disease management, healthcare utilization, and overall health outcomes relative to existing alternatives.

The rough monthly operating costs of the nationwide telemedicine system are 250,000 USD. This exceeds the rough monthly GP practice maintenance costs of 160,000 USD. It also significantly surpasses the calculated rounded 63,000 USD per month budget gap between the current and ideal care status. However, these costs may be necessary to improve health care. As mentioned above, the problem of unfilled practices is not solely financial. GPs have not filled the positions in question despite the extra 57,000 USD in allowances that were available. Also, it must be mentioned that specialized healthcare services are also delivered by the established telemedical system, along with ease of logistics with simple relocation of MHSCs, which provides further added value to the program. In addition, the telemedicine system described is not yet operating at full capacity, so the calculated differences are expected to change in favor to the telemedicine system. Due to the early state of the data collection, the financial analysis might be limited and need further throughout evaluation in the future.

The centralized logistics, combined with unified and rigorously controlled data collection, directly address challenges highlighted in previous studies, ensuring reliable and continuous monitoring of project outcomes and deliverables^5,6,31^. The research team plans further enhancements, focusing on the development of the data repository, software functionality, and the more effective wireless integration of POCT devices and telemedicine instruments.

Despite the promising results, this study has limitations, primarily the short duration of the intervention, which may have constrained the ability to detect significant health improvements. While initial findings indicate improvements in healthcare accessibility, further longitudinal studies are required to evaluate the durability of these effects and their impact on chronic disease management and health behaviors. The scalability and long-term sustainability of the telemedicine model also remain key considerations. Although the intervention successfully demonstrated the feasibility of mobile healthcare services in underserved areas, its applicability to larger populations, different healthcare settings, or urban environments with varying socio-economic conditions requires further investigation.

Another important limitation is the technological and infrastructural barriers in rural areas. The effectiveness of telemedicine services depends not only on the availability of mobile healthcare units but also on reliable internet connectivity and digital literacy among healthcare providers and patients. In some locations, inconsistent network coverage and limited familiarity with telemedicine platforms may have impacted the system’s efficiency. Addressing these disparities through targeted investments in digital infrastructure and workforce training will be essential for ensuring equitable access to care. Additionally, only a limited set of the implemented devices were used regularly, which, in line with previous studies, may be linked to healthcare workers’ restricted digital skills^39^. To enhance usability, further training programs will be necessary to facilitate the seamless integration of telemedicine technologies into routine practice.

As a next step, the research team aims to continue the intervention, based on recent good practices, broadening the scope with point-of-care obstetric ultrasound examinations and dermatology consultations and extending data collection^40–42^. In parallel, healthcare workforce training is planned to improve the technical competencies required for telemedicine-based care, which could mitigate some of the initial challenges observed. Future research should also include detailed cost-effectiveness analyses, assessing whether this model remains financially viable within different healthcare financing structures. Furthermore, continuous monitoring of patient satisfaction and provider feedback will be critical in refining service delivery and improving long-term adoption rates.

By addressing these challenges and expanding upon the initial findings, this telemedicine system has the potential to serve as a scalable and sustainable model for improving healthcare accessibility in underserved regions, while also informing broader telemedicine applications in different healthcare settings.

## Conclusion

Mobile clinics equipped with state-of-the-art telemedicine technology offer a promising solution to the healthcare challenges faced by rural and remote areas. This study introduces a nationwide telemedicine program in Hungary, targeting regions with limited access to regular healthcare services. The six-month pilot project included 1,889 patients eligible for healthcare services through public insurance, comprising both adults and children. This innovative approach featured unified and centralized logistics, coordination, data collection, and data management, ensuring seamless integration into the existing healthcare system.

The system successfully delivered both primary and specialized secondary care, mitigating the effects of general practitioner shortages and improving healthcare accessibility in the intervention areas. A notable reduction in referral rates suggests that telemedicine-supported care can alleviate pressure on traditional healthcare facilities. High patient satisfaction and provider acceptance further highlight the feasibility of integrating telemedicine solutions into rural healthcare. While patient outcomes in chronic diseases such as hypertension and diabetes showed positive trends, these changes did not reach statistical significance, presumably due to the short follow-up period, reinforcing the need for longitudinal and prospective studies to assess long-term impacts.

Moving forward, the research team aims to expand the intervention, incorporating additional diagnostic capabilities and specialist consultations while further analyzing cost-effectiveness and scalability. By addressing infrastructural and technological challenges, telemedicine has the potential to serve as a scalable and sustainable model for improving healthcare access in underserved areas, with broader implications for healthcare systems worldwide.

## Electronic supplementary material

Below is the link to the electronic supplementary material.

Supplementary Material 1

## Data Availability

The datasets generated and analyzed during the current study are available from the corresponding author on reasonable request. Due to the sensitive nature of the patient data involved, sharing is subject to compliance with participant confidentiality and ethical considerations. Researchers interested in accessing the data may request it from the corresponding author, Dr. János Sándor (janos.sandor@med.unideb.hu), with appropriate justification and agreements to use the data for non-commercial purposes. All data requests will be evaluated in line with the ethical approval granted for this study (ETT–TUKEB; BM/9240–3 /2023).
