# Supplementary material for: Bridging healthcare gaps through specialized mobile healthcare services to improve healthcare access and outcomes in rural Hungary
Source: Sci Rep. 2025 Apr 12;15:12692. doi: 10.1038/s41598-025-97447-9 (PMC11993730; doi:10.1038/s41598-025-97447-9)
Supplement: Supplementary file 1 — Supplementary Material 1 [file 41598_2025_97447_MOESM1_ESM.pdf]

Supplementary Table S1 - Control Locations. *This table lists the control locations included in the study. These municipalities were selected based on their similar geographic and socio-economic characteristics to the Programme areas, but without access to Maltese Health Points. The table provides the names of the settlements used for control comparisons in the study.*

Versend	Arlo	Kokad	Szirak	Tiszaeszlar
Mindszentgodisa	Borsodbota	Konyar	Tatarszentgyorgy	Tiszadada
Alsoszentmarton	Csernely	Hajduszovat	Istvandi	Tiszadob
Egyhazasharaszti	Domahaza	Bagamer	Kastelyosdombo	Tarpa
Old	Farkaslyuk	Ujleta	Lakocsa	Beregdaroc
Siklosnagyfalu	Hangony	Sap	Dravagardony	Olcsva
Cserdi	Jardanhaza	Szerep	Bolho	Aranyosapati
Sumony	Jakfalva	Kerecsend	Iharos	Gemzse
Somogyapati	Kelemer	Hevesaranyos	Iharosbereny	Gyure
Somogyhatvan	Sajonemeti	Gyongyosoroszi	Gyugy	Ilk
Zador	Zadorfalva	Halmajugra	Somogyvamos	Tiszaadony
Dencshaza	Gyorgytarlo	Nagyfuged	Csokoly	Tiszakerecseny
Kunagota	Viss	Vecs	Beleg	Tiszavid
Geszt	Felsoregmec	Tarnabod	Gorgeteg	Tuzser
Damoc	Kovacsvagas	Atany	Kisbajom	Eperjeske
Ricse	Prugy	Komlo	Labod	Tiszabezded
Tiszakarad	Taktakenez	Tiszanana	Nagykorpad	Tiszamogyoros
Zemplenagard	Taktaszada	Erk	Otvoskonyi	Kisvejke
Semjen	Girincs	Tarnaors	Rinyaszentkiraly	Gyulaj
Tiszacsermely	Hejoszalonta	Tarnaszentmiklos	Szabas	Kocsola
Boldogkoujfalu	Kesznyeten	Tarnazsadany	Tarany	Sarpilis
Goncruszka	Nemesbikk	Erdokovesd	Tengod	Erteny
Vizsoly	Taktabaj	Szentdomonkos	Nagyecsed	Furged
Felsodobsza	Baks	Tarnalelesz	Paszab	Magyarkeszi
Vilmany	Csanadalberti	Tiszabo	Tiszabercel	Nagyszokoly
Pere	Kiralyhegyes	Tiszabura	Szekely	Ozora
Sajokaza	Nagyer	Tomajmonostora	Tiszarad	Pari
Felsotelekes	Sarkeresztur	Csepa	Beszterec	Pusztamiske
Arokto	Told	Tiszaderzs	Kekcse	Nagydem
Tiszatarjan	Bojt	Tiszaigar	Retkozberencs	Dioskal
Saly	Hencida	Hugyag	Tiszakanyar	Mikekaracsonyfa
Gesztely	Berekboszormeny	Csesztve	Ujkenez	
Korom	Korosszakal	Matraverebely	Bokony	
Onod	Mezosas	Ecseg	Nagycserkesz	
Sajopetri	Pocsaj	Kallo	Timar	

Supplementary Table S2 - Difference in outpatient specialist attendance. *This table compares the outpatient specialist attendance rates between the Programme areas, control areas, and national averages. Standardized indicator values with 95% confidence intervals are presented to highlight significant differences, if any, between the different regions.*

**Area**	**Raw indicator value for general practitioners (%)**	**Standardised indicator value in general practice districts (% [95% CI])**	**Standardised indicator value deviation from the national data**	**Standardised indicator value deviation from control general practitioner data**
**National**	1,68	100,15 [99,59-100,71]	-	
**Control Area**	1,45	95,84 [92,58-99,18]	significantly lower	-
**Programme**	1,40	91,5 [83,62-99,94]	not significant	not significant
Zalakomar	1,47	91,91 [75,66-110,00]	not significant	not significant
Hirics	1,41	91,10 [70,99-116,78]	not significant	not significant
Litke	1,09	69,16 [53,26-88,31]	significantly lower	significantly lower
Szalonna	1,31	90,72 [70,79-115,57]	not significant	not significant
Nyirkata	1,54	103,45 [89,25-119,90]	not significant	not significant

Supplementary Table S3 – BMI in the examined Population. *This table provides data on the Body MAss Index (BMI) of patients in the Programme areas. It shows the number of individuals with a BMI over or under 24.99 kg/m², grouped by each district involved in the Programme. These values are used to assess the prevalence of obesity within the examined population.*

	**Over 24.99 kg/m²**	**Under 24.99 kg/m²**	**All examined patient**
**Programme**	701	376	1077
Zalakomar	129	60	189
Hirics	103	72	175
Litke	172	86	258
Szalonna	107	64	171
Nyirkata	190	94	284

Supplementary Table S4 - Abdominal circumference in the examined population. *This table presents the abdominal circumference measurements of patients in the Programme areas, indicating how many patients exceeded the normal threshold values (88 cm for women and 102 cm for men). Data is provided for each district.*

	**Above threshold**	**Under threshold**	**All examined patient**
**Programme**	589	309	898
Zalakomar	113	47	160
Hirics	95	43	138
Litke	146	77	223
Szalonna	90	60	150
Nyirkata	145	82	227

Supplementary Table S5 – Regular smokers in the examined population. *This table lists the number of smokers and non-smokers in the Programme population, grouped by district. The table offers insight into the prevalence of smoking in the examined population compared to national averages.*

	**Smokers**	**Nonsmokers**	**All examined population**
**Programme**	646	625	1271
Zalakomar	86	118	204
Hirics	89	67	156
Litke	150	135	285
Szalonna	147	123	270
Nyirkata	174	182	356

Supplementary Table S6 - Regular alcohol consumers in the examined population. *This table shows the number of regular drinkers and non-drinkers in the Programme population. It provides data on alcohol consumption patterns in each district, comparing them with national values.*

	**Regular drinkers**	**Nondrinkers**	**All examined population**
**Programme**	475	735	1210
Zalakomar	77	107	184
Hirics	39	106	145
Litke	119	157	276
Szalonna	99	153	252
Nyirkata	141	212	353

Supplementary Material M1 - Patient feedback questionnaire. *This material contains the patient feedback questionnaire used in the study to gather information on patient experiences with the telemedicine services provided. The questionnaire includes questions about patient satisfaction, the perceived quality of care, and overall experience during telemedicine consultations.*

1. **What is your gender?**
    - Male
    - Female
    - Other
2. **What is your age?** *(Please enter a whole number, e.g., 43)*
3. **What is your highest level of education?**
    - ○ Less than 8 years of elementary education
    - ○ Completed 8 years of elementary education
    - ○ High school diploma
    - ○ Vocational qualification
    - ○ Higher education (college or university)
4. **In which municipality did your care take place?** *(Please write only the name of the place, e.g., Gacsaly, Hirics)*
5. **What type of examination did you come to us for?**
    - ○ General check-up or screening
    - ○ Acute complaint
    - ○ Follow-up after a previous examination or ongoing care
    - ○ Other
6. **How would you describe your general health condition?**
    - ○ Very good
    - ○ Good
    - ○ Satisfactory
    - ○ Poor
    - ○ Very poor
7. **How has your health changed in the past year?**
    - Improved
    - Remained the same
    - Worsened
8. **In your opinion, how much can you do for your health?**
    - A great deal
    - Quite a lot
    - A little
    - Nothing
9. **How much do you think you are currently doing for your health?** *(Please consider your diet, alcohol consumption, smoking habits, and sleep patterns.)*
    - A great deal
    - Quite a lot
    - A little
    - Nothing
10. **Were you happy to come to the Maltese Charity Service's clinic today?**

- Yes
- Mostly
- No

11. **Did you discover anything new about your health condition?**
    - No
    - Yes, my health is better than I expected
    - Yes, my health is worse than I expected
12. **Was the consultation with the doctor and nurse understandable to you?**
    - ○ Completely understandable
    - ○ Mostly understandable
    - ○ Less understandable
    - ○ Not understandable
13. **During the examination, you spoke with the doctor via internet video connection. How did this feel to you?**
    - ○ Natural
    - ○ Exciting and new
    - ○ Acceptable
    - ○ Uncomfortable
    - ○ Other: ________
14. **You encountered several new, modern examination tools during your visit. Overall, how much do you trust these devices?**
    - ○ Completely
    - ○ Mostly
    - ○ Slightly
    - ○ Not at all
15. **Overall, are you satisfied with the care you received from us?**
    - ○ Completely satisfied
    - ○ Mostly satisfied
    - ○ Slightly dissatisfied
    - ○ Completely dissatisfied
16. **Do you have any unanswered questions?**
    - ○ Yes, unfortunately
    - ○ Maybe
    - ○ No, all my questions were answered
17. **Overall, how confident are you that your health will improve following our care?**
    - ○ Very confident
    - ○ Somewhat confident
    - ○ Not very confident
    - ○ Not confident at all
18. **Would you be willing to return to us for future examinations?**
    - ○ Definitely yes
    - ○ Probably yes
    - ○ Probably not
    - ○ Definitely not
19. **Would you recommend our medical services to your family and friends?**

- ○ Definitely yes
- ○ Probably yes
- ○ Probably not
- ○ Definitely not

Supplementary Material M2 - Doctor feedback questionnaire. *This material includes the feedback questionnaire distributed to doctors participating in the telemedicine Programme. It collects data on their experiences with telemedicine, the perceived quality of care compared to in-person consultations, and their overall satisfaction with the system.*

1. **Patient's full name:**
   Answer type: Required text input.
2. **Examination date:**
   Answer type: Date picker.
3. **Location:**
   Answer type: Dropdown list of locations.
4. **Social Security Number:**
   Answer type: Required text input.
5. **Purpose of the visit (Why did the patient come?):**
   Answer type: Dropdown list: Screening / Follow-up / Acute care / Administrative task.
6. **Estimated time of patient care (in minutes) during teleconsultation:**
   Answer type: Required number input.
7. **Administrative time related to patient care (in minutes) outside the consultation:**
   Answer type: Required number input.
8. **Suggestions for improving care (tools, professional guidelines, methodology, etc.):**
   Answer type: Optional text input.
9. **If you had seen the patient in person, would you have provided less, the same, or more care?**
   Answer type: Dropdown: Less / The same / More.
10. **In your opinion, how easy would it have been for the patient to access other medical care for the same issue?**
    Answer type: Detailed feedback required.
11. **Do you consider the case professionally educational?**
    Answer type: Dropdown: Yes / No.
12. **Please provide detailed feedback regarding the assistant's work (use of tools, professionalism, communication, etc.):**
    Answer type: Optional text input.
13. **To what extent did you feel safe professionally or personally during the consultation?**
    Answer type: Dropdown: Fully / Mostly / Partially / Not at all.
14. **Overall, how did you perceive the examination situation?**
    Answer type: Dropdown: 5 (very good) to 1 (very poor).
15. **Is there anything else you consider important about this examination event?**
    Answer type: Optional text input.
